# Developmental neurotoxicity of monocrotophos and lead is linked to thyroid disruption

**DOI:** 10.14202/vetworld.2016.133-141

**Published:** 2016-02-08

**Authors:** B. Kala Kumar, A. Gopala Reddy, A. Vamsi Krishna, S. S. Y. H. Quadri, P. Shiva Kumar

**Affiliations:** 1 Department of Veterinary Pharmacology & Toxicology, College of Veterinary Science, Sri P.V. Narsimha Rao Telangana State University for Veterinary, Animal and Fishery Science, Hyderabad - 500 030, Telangana, India; 2 Department of Bio-technology, Ministry of Science & Technology, New Delhi, India; 3 Department of Pathology, National Institute of Nutrition (ICMR), Hyderabad, Telangana, India; 4 Sri P.V. Narsimha Rao Telangana State University for Veterinary, Animal and Fishery Science, Hyderabad - 500 030, Telangana, India

**Keywords:** behavioral alterations, developmental neurotoxicity, lead, monocrotophos, thyroid disruption

## Abstract

**Aim:**

A role of thyroid disruption in developmental neurotoxicity of monocrotophos (MCP) and lead is studied.

**Materials and Methods:**

A total of 24 female rats after conception were randomized into four groups of six each and treated as follows: Group I - Sham was administered distilled water orally. Group II - A positive control was administered methyl methimazole at 0.02% orally in drinking water. Group III - MCP orally at 0.3 mg/kg and Group IV - Lead acetate at 0.2% orally in drinking water. The drug was administered from gestation day 3 through post-natal day 21 in all the groups. Acetylcholinesterase (AChE) inhibition, thyroid profile (thyroid stimulating hormone, T_3_ and T_4_), neurodevelopment (brain wet weights, DNA, RNA and protein), and neurobehavioral (elevated plus maze, photoactometry, and Morris water maze) parameters were assessed in pups. A histopathology of thyroid of dams and brain of progeny was conducted.

**Results:**

Inhibition of AChE was <20%. Thyroid profile decreased in the treatment groups. Neurodevelopmental and neurobehavioral parameters did not reveal any significant changes. Thyroid architecture was affected significantly with MCP and lead. Cortical layers too were affected. The three layers of cerebellum either had abnormal arrangement or decreased cellularity in all treated groups relating to thyroid disruption.

**Conclusion:**

MCP and lead might have affected the development of cerebrum and cerebellum via thyroid disruption leading to developmental neurotoxicity.

## Introduction

The potential harmful chemicals or substances, such as heavy metals, pesticides, and hydrocarbons, are dumped either or released into the water bodies [1]. When these pollutants flow into water bodies in a higher concentration than permissible limits then these result in the form of heavy mortalities of all life form residing in those aquatic systems such as fish and shellfish, etc., while in a lower concentration these lead to bioaccumulation of these pollutants and ultimately go through the food web to human beings. These pollutants can also alter other hormonal processes of fish like the development of bones and proper thyroid functioning. Dimethoate and lambda-cyhalothrin showed the lethal effect on thyroid hormone of *Labeo rohita* [2]. Fetal exposure to environmental chemicals could affect the development of nervous system. In this chemical age, certain of the developmental defects do not have a definite etiology and the only pointer could be exposure during development that too at a critical time. With the rise in the use of pesticides, surfacing of behavioral disorders became common. A majority of children suffer from neurodevelopmental disorders and exposure to xenobiotics has been identified as one of the risk factors. About 8 million children suffer from one or other mental disorders, and 1.1 million are exposed to organophosphate (OP) insecticides above the safety levels. One of the facets of OP toxicity is chronic OP-induced neuropsychiatry disorders. While researching on the developmental neurotoxicity of OPs, especially chlorpyrifos (CPS), their cholinesterase-independent actions came into the fore and have surpassed the receptor level and are lingering at the cell signaling mechanisms. One aspect that has been attempted albeit on a lesser scale is the interference of endocrine mechanisms by OPs that could contribute to the existing neurotoxicity on in-utero exposure.

Lead (Pb) has been implicated in a variety of behavioral disorders since its use in 1900 as leaded gasoline and other forms. Although a unified mechanism of action has been elusive, it is believed to be the outcome of a yet to be identified abnormal process or toxic insult in-utero or during early post-natal life. The subsequent challenge in the adult life of the exposed fetus could cause behavioral abnormalities.

Maternal thyroid hormone availability is crucial for the development of fetal brain [3] and influence the expression of genes in neurogenesis, gliogenesis, maturation, differentiation and migration. All these developmental activities are time-dependent, and any delay could literally compromise the cytoarchitecture of the brain and is manifested as abnormal behavior.

Against this backdrop, the present study was proposed to link the developmental neurotoxicity of monocrotophos (MCP) (an extensively used OP pesticide) and lead (a ubiquitous heavy metal and environmental pollutant) with thyroid disruption.

## Materials and Methods

### Ethical approval

This study was conducted after approval by the Research Committee and Institutional Animal Ethics Committee.

### Experimental design

Rats of Sprague-Dawley strain were procured from National Centre for Laboratory Animal Sciences, National Institute of Nutrition, Hyderabad and maintained under standard conditions. Institutional Animal Ethics Committee, College of Veterinary University, Rajendra Nagar, permission was obtained before the conducting of the experiment and standard humane procedures were adopted. MCP, (purity 77.4%) was supplied by Hyderabad Chemicals Pvt. Ltd., India as a gratis sample. Methyl methimazole (MMI) (METHIMEZ 10 mg, Sun Pharma Pvt. Ltd.), lead acetate (PbAc), and other chemicals used in the experiment were of analytical grade.

Female rats were mated overnight, and the presence of sperm in the vaginal smear was considered as positive for conception (gestational day [GD] zero). 24 females after conception were randomized into four groups of six each and treated as follows: Group I - Sham was administered distilled water orally (5% of body weight). Group II - a positive control was administered MMI at 0.02% orallyas sole source of drinking water. Group III - MCP orally at 0.3 mg/kg b.wt and Group IV - PbAc at 0.2% orallyas sole source of drinking water. The drug was administered from GD3 through post-natal day (PND) 21 in all the groups.

### Sample collection and preparation

On GD 14, whole blood was collected from dams for estimation of erythrocyte acetylcholinesterase (AChE) [4] in control and MCP treatments. Further, serum was used for estimation of thyroid profile (thyroid stimulating hormone [TSH], T_4_ and T_3_) in all the groups by radio immunoassay employing Dia Sorin S.P.A. kits, USA. The radio-labeling was detected by automated clinic gamma counter; LKB Wallace, Finland. On PND 7, brains of the pups were rapidly dissected and neurodevelopmental parameters (brain wet weight, DNA, RNA, protein) assessed. Few brains were fixed in 10% formalin for ­histopathology examination (HPE). DNA was extracted [5] and estimated by UV7500 UV-VIS Spectrophotometer of Techcomp, Ltd., Hong Kong, whereas, RNA was quantified by Trizol method and protein by biuret method [6]. On the day of weaning, i.e., PND 21, mothers were sacrificed and thyroids collected for HPE [7]. Neurobehavioral studies Morris water maze (MWM), elevated plus maze (EPM), photoactometry were conducted in 21-day-old F_1_ rats.

### Statistical analysis

The data were subjected to statistical analysis by applying one-way ANOVA using Statistical Package for Social Sciences version 15.0. Differences between means were tested using Duncan's multiple comparison test and a significance level was set at 0.05.

## Results

Erythrocyte AChE (%) activity in dams was measured in control and MCP-treated groups only. MCP treatment showed 20.38% of AChE inhibition (Table-1).

**Table-1:** AchE inhibition in dams and neurodevelopment parameters in 7-day-old rats.

Group	AchE* activity (%)	Wet weight of brain in (g)	DNA(μg/g)	RNA(μg/100 mg)	Protein concentration (%)
I control	0.0	1.339±0.020^b^	0.0313±0.0065^a^	65.50±0.80^ab^	1.00±0.23^ab^
II MMI	-	1.309±0.328^b^	0.0183±0.0017^a^	72.50±5.18^b^	1.42±0.16^b^
III MCP	20.38	1.450±0.429^a^	0.0251±0.0029^a^	57.00±5.23^ab^	0.73±0.12^a^
IV PbAc	-	1.284±0.055^b^	0.0257±0.0031^a^	63.83±0.54^ab^	1.20±0.07^ab^

* Inhibition of AchE (μM of Ach hydrolyzed by 1 g of brain tissue at 37°C in 20 min). Values are mean±SE (n=6); one-way ANOVA (SPSS); p<0.05. Means with different alphabets as superscripts differ significantly (different alphabets denotes there is some significant difference between them). AChe=Acetylcholinesterase, MMI=Methyl methimazole, MCP=Monocrotophos, PbAc=Lead acetate, SE=Standard error

### Neurodevelopment parameters

Wet weight, DNA, RNA and protein were the parameters assessed to evaluate developmental neurotoxicity in rat pups on PND 7 (Table-1).

The mean wet weight of brains (g) of the progeny in Group I, II, and III were 1.339±0.02, 1.309±0.32, and 1.284±0.05, respectively.There appeared to be no statistical significance between and the treatments. However, in Group IV a statistically significant (p<0.05) increase in brain weights 1.450±0.42 g was noted.

The mean concentration of DNA (μg/g) in brain tissue was 0.0313±0.0065, 0.0183±0.0017, 0.0251±0.0029, and 0.0257±0.0031μg/g of in Groups I, II, III, and IV, respectively. There was a non-significant difference in the concentration of DNA between control and the treated groups. MMI group recorded the least concentration of DNA.

The concentration of RNA in the brain (μg/100 mg of brain matrices) in various groups was 62.50±0.80, 72.50±5.18, 57.00±5.23, and 63.83±0.54, respectively, in Groups I through IV. Group II had the highest concentration of RNA while Group V recorded the lowest when compared to control. The remaining Groups (III and IV) registered a decrease in the levels of RNA as compared to control, but the decrease was non-significant.

Protein % in the whole brain showed a significant difference (p<0.05) in MMI and MCP when compared to control. The protein % was highest in MMI (1.42±0.16), whereas in MCP it was lowest (0.73±0.12).

Thyroid assay included the measurement of TSH, T_3_ and T_4_ by radio immunoassay on the 14^th^ day of gestation (Table-2).

**Table-2:** Thyroid profile of dams and photoactometry in F_1_ generation rats.

Group	TSH (μU/ml)	T_3_ (ng/ml)	T_4_ (μg/dl)	Photoactometry activity/minute
I control	0.098±0.012^a^	0.9835±0.105^a^	2.285±0.13^b^	103.16±6.33^a^
II MMI	0.107±0.009^a^	0.862±0.034^a^	2.1151±0.12^b^	81.66±7.27^a^
III MCP	0.129±0.014^a^	1.0301±0.091^a^	2.1955±0.14^b^	98.83±11.81^a^
IV PbAc	0.119±0.017^a^	1.0426±0.105^a^	2.7588±0.18^a^	116.00±18.05^a^

Values are mean±SE (n=6); one-way ANOVA (SPSS); p<0.05. Means with different alphabets as superscripts differ significantly (different alphabets denotes there is some significant difference between them). TSH=Thyroid stimulating hormone, MMI=Methyl methimazole, MCP=Monocrotophos, PbAc=Lead acetate, SE=Standard error

TSH (μU/ml) concentration increased in treatment groups as compared to control.Such an increase was apparent but statistically non-significant in Groups III and IV, except MMI-treated Group (II) that showed statistically significant (p<0.05) decrease in TSH concentration.

The concentration of the triiodothyronine (T_3_) (ng/ml) decreased significantly (p<0.05) in Group II (0.862) as compared to control (0.983). MCP and PbAc treated Groups (III and IV) had a slight non-significant increase in T_3_ concentration when compared to control.

The mean concentration of thyroxine (T_4_) (μg/dl) T_4_ was 2.11±0.12 and 2.19±0.14, respectively, in Groups I and III. All the groups had the T_3_ concentration on par with control, and there was no statistically significant difference. Group II (MMI) had a decreased concentration of T_4_ while Group IV (PbAc) showed an increase in T_4_ concentration (2.75±0.18) as compared to control.

The motor activity of the progeny of the treated rats was determined by photoactometer (activity/min). The activity of the rats in the control group was 103.16±6.3 with a decrease in groups MMI and MCP (81.66±7.2 and 98.83±11.8) respectively. In the case of PbAc, the activity was slightly higher than in control group. However, the differences between control and other treatments were statistically non-significant (Table-2).

The anxious behavior of the experimental rats was assayed by EPM and the results are presented in Table-3. A number of entries in open arm: It is an indicator of the anxious behavior of rats. The number of entries into the open arm needs to be less in Group I rats. The control group rats made a mean of 1.35±0.51entries, whereas rats of Groups II, III and IV had a mean entry of 1.9±0.67, 1.5±0.53 and 1.5±0.75, respectively, which was higher than control. A number of entries in closed arm: The normal behavior of rats is that they prefer to stay in closed spaces. Control rats made a mean of 3.70±0.51 entries while Group II rats had less number of entries (2.80±0.67 entries). The mean entries into the closed arm were more in Group III and Group IV was 4.73±0.55 and 4.00±0.75 respectively. Duration of time spent (seconds) in the center of maze: Animals of control group spent about 20.23±19.87 s in the center of the maze. All other treated groups spent more time compared to control group. The order of increased time spent was 39.00±25.91 s (Group II), 31.22±27.31 s (Group III), and 23.00±28.97 s (Group IV). Thus, MMI treated rats spent more time in the center of the maze. However, there was no significant difference between control and treated groups. Duration of time spent (seconds) in open arm: MMI and MCP groups (92.90±25.91 s and 78.22±27.31 s, respectively) spent more time, whereas, PbAc (46.62±28.97) spent less time when compared to control (63.00±19.87 s), and were without statistical significance.Duration of time spent (seconds) in the closed arm: Sham rats spent 199.41±19.87 sin closed arm. Groups II and III (162.70±25.91 s and 182.77± 27.31 s, respectively) spent lesser time in closed arm as compared to sham. Rats of Groups IV (245.00±28.99 s) spent more time in closed arm than the control group. Although apparent differences existed, they were statistically non-significant. Total distance travelled: Control group traversed 416.87 cm, whereas Groups II, III and IV travelled 500±64.22, 487.5±108.29 and 443.33±54.13 cm, respectively, which is higher than control.

**Table-3:** Performance of first generation adult rats on EPM.

Group	Number of entries		Percent of entries		Duration of time spent in seconds			Total distance travelled (cm)
		
	Open arm	Closed arm	Open arm	Closed arm	Centre of the maze	Open arm	Closed arm
I control	1.35±0.51	3.70±0.51	26.7	73.26	20.23±19.87^a^	63.00±19.87^a^	199.41±19.87^b^	416.87±52.85
II MMI	1.90±0.67	2.80±0.67	40.42	59.57	39.00±25.91^a^	92.90±25.91^a^	162.70±25.91^b^	500.00±64.22
III MCP	1.50±0.53	4.73±0.55	24.07	75.23	31.22±27.31^a^	78.22±27.31^a^	182.77±27.31^b^	487.50±108.29
IV PbAc	1.50±0.75	4.00±0.75	27.27	72.72	23.00±28.97^a^	46.62±28.97^a^	245.00±28.97^b^	443.33±54.13

Values are mean±SE (n=6); one way ANOVA (SPSS); p<0.05. Means with different alphabets as superscripts differ significantly (different alphabets denotes there is significant difference between them). EPM=Elevated plus maze, MMI=Methyl methimazole, MCP=Monocrotophos, PbAc=Lead acetate, SE=Standard error

The results of escape latency (EL) and probe trial of MWM are expressed in seconds and presented in Table-4. The data pertaining to the EL revealed that control group took 44.87±6.56 s to identify the platform. Other treated groups had taken considerably more time in identifying the platform. Groups IV (59.75±0.25 s), II (58.00±2.00 s), and III (57.25±2.75 s) were slow in detecting the platform. The same pattern existed in the second learning trial, but for a reduction in 14 s in EL2 in the control group as compared to EL1. Groups III and IV had identified the platform early compared to EL1. In EL3, the sham rats identified the platform in 33.37±7.50 s, while Group III (38.5±8.38 s) took lesser time in identifying the platform as compared to Group II (57.00±3.00) and Group IV (42.62±8.55). There was no statistically significant difference between control and treated groups, although apparent differences exist. The probe trial is depicted in Table-4. Control Group (I) detected the hidden platform in 34.25±7.47 s. Group III in 32.62±8.39 s, whereas Groups IV (52.25±5.35 s) and II (50.37±7.03 s) took a longer time to detect the platform than control and MCP with non-significant difference. Total distance travelled: The distance travelled by the animals is depicted in the Table-4. This indicates the primary effort of the animal to explore a solution to the existing problem. There was a significant difference (p<0.05) between the control and the treated groups. Rats of Group II were the most inactive, at times the rats stayed at the place of entry and were not willing to move or explore. This was followed by Group IV, which expressed thigmotaxis rather than spending most of the time at the center of the pool, a property constantly observed with control group. Group III displayed significant alertness in finding the platform next to control group.

**Table-4:** Performance of first generation adult rats on MWM.

Groups	EL (in seconds)			Probe trial (seconds)	Total distance travelled (cm)

	EL1	EL2	EL3		
I control	44.87±6.56^a^	30.12±7.65^a^	33.37±7.50^a^	34.25±7.47^ab^	1462.50±17.87^d^
II MMI	58.00±2.00^a^	40.12±7.71^a^	57.00±3.00^a^	50.37±7.03^b^	284.17±8.79^a^
III MCP	57.25±2.75^a^	31.5±6.68^a^	38.5±8.38^a^	32.62±8.39^ab^	989.17±6.37^c^
IV PbAc	59.75±0.25^a^	49.62±5.92^a^	42.62±8.55^a^	52.25±5.35^b^	687.50±6.15^b^

Values are mean±SE (n=6); one-way ANOVA (SPSS); p<0.05. Means with different alphabets as superscripts differ significantly (different alphabets denotes there is some significant difference between them). EL=Escape latency, MMI=Methyl methimazole, MCP=Monocrotophos, PbAc=Lead acetate, SE=Standard error, MWM=Morris water maze

### Histopathology

**Figure-1:**
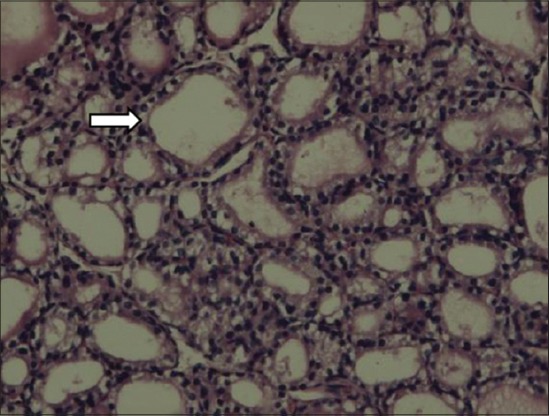
Empty follicles, shrunken in size, stromal abundance in thyroid gland of dams (Group III) (H and E).

**Figure-2:**
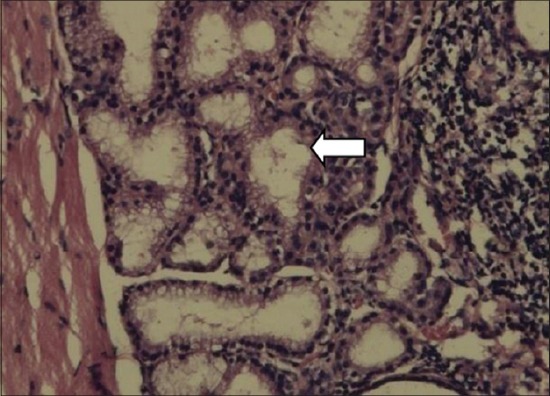
Empty follicles. Disrupted cytoarchitecture in thyroid gland of dams (Group IV) (H and E,×200).

In thyroid gland of dams, the follicles in the control group were large, circular and oval in a shape filled with homogenous, eosinophilic colloid material. The interlobular stroma was poorly defined. The follicular cells were flattened. Among the treatment groups, MMI, MCP and PbAc rats microfollicles were visible, had an irregular shape, absolutely empty with no colloid material and dense stroma appeared between the lobules (Figures-1 and 2).

**Figure-3:**
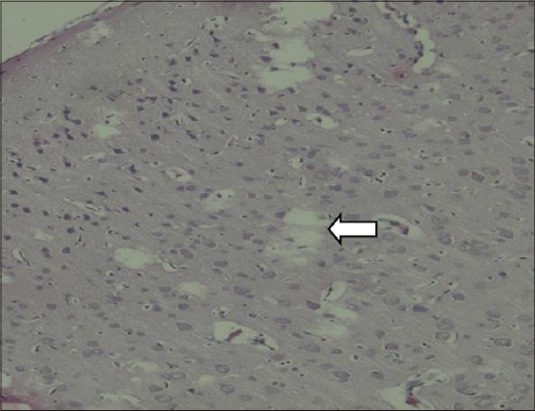
Decreased external pyramidal cells and vacoulation in brain of pups (Group III) (H and E,×200).

**Figure-4:**
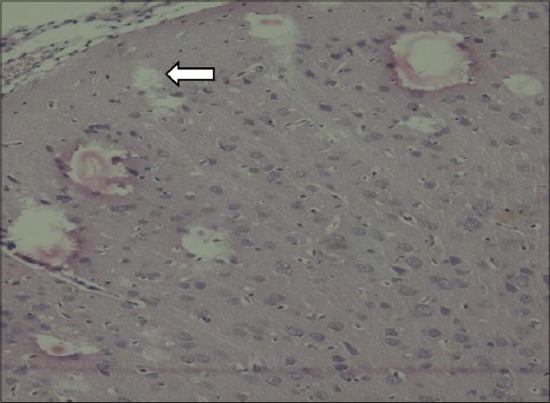
Vacoulation in cortical layers with reduced external pyramidal layer in brain of pups (Group IV) (H and E,×200).

**Figure-5:**
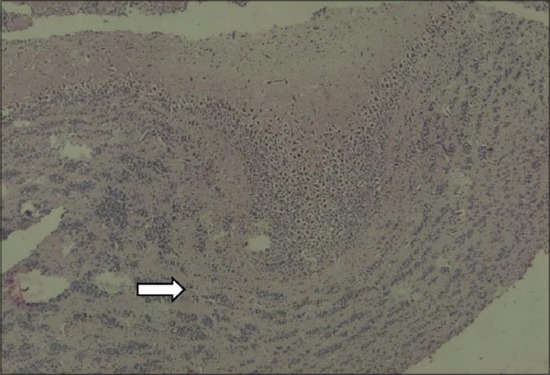
Disorganized Purkinje cells and granular layer is loosely arranged in brain of pups (Group III) (H and E,×200).

**Figure-6:**
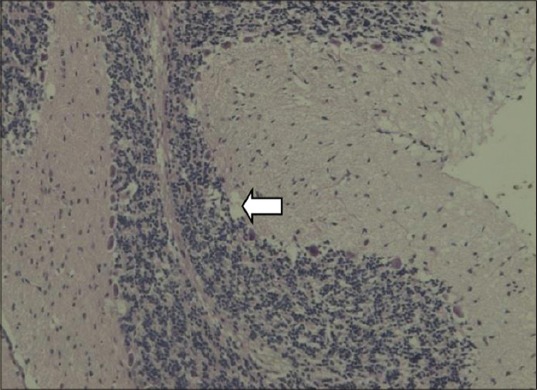
Focal absence of Purkinje cells in brain of pups (Group IV) (H and E,×200).

In the brain of F_1_ progeny, the cerebral cortical layers showed a decrease in cellularity in all the treated groups as compared to control (Figures-3-6).

## Discussion

The developmental neurotoxicity of MCP and lead on maternal thyroid disruption and fetal outcome in Sprague-Dawley rats was studied.

### AChE activity

Erythrocyte AChE activity in MCP treated animals was inhibited upto 20.38% as compared to control, and the inhibition was not significant at the dose employed. Greater than 20% inhibition causes developmental neurotoxicity, whereas 70% inhibition is said to produce cholinergic hyperstimulation [8]. The dose of 0.3 mg/kg was selected to have the minimum or no effect on AChE inhibition. This was done with an aim to specifically evaluate the non-cholinergic mechanisms involved in the developmental neurotoxicity. The inhibition of the enzyme by MCP was dose-dependent in day-old chick brain [9], in mice [10] and Wistar rats. The latter two investigators employed the same dose (0.3 mg/kg) as in this experiment and have reported significant inhibition of AChE; such a variance could be due to the difference in species and strain employed.

Wet weight of brains was recorded in pups on PND 7. Syed and Shafiullah [11] employed the same dose of MMI as in our study, and no effects were observed on the organ weights of fetuses. CPS at 5 mg/kg/day administered perinatally from GD 10-PND10 decreased neonatal brain weights [12]. However, an increase in brain weights could probably be due to the higher body weights of the pups in MCP group.

DNA→RNA→ protein sequence, the vital lead for the function of the cell and gene expression, is said to be affected by environmental factors, especially contaminants like heavy metals, pesticides, polychlorinated biphenyls, hormones, etc., [13]. PbAc*in vitro* induced single and double DNA strand breaks and DNA-protein cross links [14]. Pesticides are known genotoxicants; hence, the DNA content of brain was estimated. DNA is a good indicator of cell number. MCP, orally, caused dose-dependent alkylation of DNA in mice but 72 h post-treatment damaged DNA was repaired as indicated by a decrease in comet tail length [15]. Diazinon exposure in zebra fish decreased DNA, RNA and total protein of liver [16] whereas, CPS damaged DNA in the liver and brain of rats [17] in a dose-dependent manner. Fetal DNA methylation patterns, as evident in a number of animal studies, have established outcomes that are carried to later life [18]. On the same lines of methylation, demethylation in the embryo also helps to remove epigenetic modifications. Thus, loss or gain of methylation is age and cell, tissue or organ dependent. Genotoxicants like cadmium and pesticides cause DNA strand breaks or fragmentation [19,20]. Poly (ADß-ribose) polymerase-1 senses breaks and promotes repair. The addition of protein too decreases the toxicity as evidenced in CPS and diazinon inhibited DNA synthesis *in vitro* in neuronotypic PC-12 cells and gliotypic C6 cells [21]. There was an apparent decrease in DNA in all the treated groups in the present work, when compared with control, but such a decrease was not significant statistically. The results are in agreement with an earlier work on day-old chicks where DNA reduction was not statistically significant. This could be due to age- and time-dependent demethylation of DNA. Furthermore, lead intoxicated neonatal rats displayed retardation of new cell formation in the cerebellum but little effect on the concentration of RNA, DNA and protein content in the developing rat brain. However, Pb at 0.1 or 1 mg/ml for 1 month in mice had decreased body weight and DNA concentration.

RNA is a link between gene and protein expression. A decrease in DNA is followed by a decrease in RNA and protein in zebra fish exposed to diazinon in hypothyroid animals [22] and in mice embryo liver treated with MCP, whereas in the present study, RNA concentration did not show statistically significant difference from the control as was DNA. RNA in MMI Group (II) was apparently the highest but not different from control. As there was no decrease in DNA content, it might be inferred that the RNA content also remained stable.

Protein was quantified to estimate the cell size of the brain of the pups. As discussed above, a fall in DNA would be subsequently reflected in the levels of RNA and protein. OPs cause adduction of proteins specific to the compound [23]. PbAc inhibited protein synthesis in cultures of rat sertoli cells *in vitro*. In the current experiment, MMI had high protein levels and MCP the lowest. MMI also had a high concentration of RNA and that might have influenced the protein expression. The difference between the MMI and MCP was statistically significant, albeit the difference between these two groups when compared to control was not significant. The present findings are in agreement with Bharani and Reddy [7] with MCP in neonate chick brain and with parathion in rats.

The most common used endpoints to monitor thyroid function currently are serum thyroid hormone levels (T_4_ and T_3_), TSH and thyroid histology [24]. Maternal T_4_ has a greater influence on early neurodevelopment because the brain receives higher levels of T_4_ that is converted subsequently to T_3_. In the present investigation, the levels of T_3_ and T_4_ were reduced but TSH was on the rise in the MMI-treated group, as a compensatory mechanism but such an increase in serum TSH is an issue of timing and could be dissociable temporarily [25].

Earlier workers have reported a dose-dependent decrease of T_4_ with aroclor 1254 in rat [26], dichlorodiphenyl dichloroethylene and dichlorodiphenyltrichloroethane in human infants [27]. Organochlorine compounds decrease or mimic the actions of thyroid hormones by interfering with HPT axis, induce thyroid metabolizing enzymes and competitively bind to transporter protein. But in PbAc-treated group, an increase in T_4_ and a normal TSH could be due to a general resistance to thyroid hormone by the receptors or inhibition of Type II deiodinase or inhibition of microsomal glucuronidation and bile elimination of T_4_ in rats. Lead is a known microsomal enzyme inhibitor, and this could have added to the increased T_4_ concentration. Such an increase in T_4_ could produce impaired cognition, lack of concentration and inattentiveness [28]. Environmental toxicants can interfere with thyroid function (thyroid-dependent) or could act on receptors without affecting the thyroid gland (thyroid-independent) and may produce adverse neurodevelopmental effects, which may not fully agree with hypothyroidism or thyroid toxicity [29]. Pb is both negatively correlated with thyroid hormones and positively correlated [30] as a function of the dose. In contrast, [31] reported no relationship between Pb and thyroid hormones. Furthermore, some of the congeners of a compound may bind to thyroid hormone receptors of a particular species of animals and the same affinity may not be displayed in other species. Thus, thyrotoxicity is dependent on dose, compound and species.

The motor response and the exploratory behavior of treated animals were measured by photoactometry. MMI animals had less exploratory behavior but PbAc group exhibited hyperactivity, although the activity did not differ significantly with control. PbAc is known to cause hyperactivity. These results are consistent with Kuriyama *et al*. [32] for propyl thiouracil (GD7-21), but in adults, a contrast of reduced locomotor activity was observed for 2 days. Locomotor activity was dependent on the dose and PND of exposure of CPS, ranging from no effect to decreased activity in rats [33] and in mice with diisopropyl fluorophosphate.

EPM determines the animal's unconditioned response to a potentially dangerous environment. A normal rodent avoids open arm for fear or anxiety and spends most of the time in closed arms, called thigmotaxis, i.e. the tendency to remain close to the walls. PbAc is said to affect the behavior of the animal and thus increase its anxiety. Hence, this might have made the animals stick to the walls. MMI-treated animals spent more time in the open arm, whereas MCP-treated animals had spent more time in closed arm infering loss of fear and anxious behavior in these two groups, respectively. The findings are consistent with Sanchez-Amate *et al*. [34], who observed anxiogenic-like response with CPS in dams during GD 9-12 and in OP pesticide applicators.

MWM is the gold standard for determining cognitive skills in animals. Changes in the central cholinergic system can be detected, and cued version is capable of revealing deficits in sensory, motor or motivational processes. Repeated acquisition (learning) and performance (memory) were assessed in all the groups. A detailed analysis of swimming patterns revealed thigmotaxis in MMI and PbAc treatments, i.e. a higher percentage of time in outer zone and lesser time in the middle zone, the zone of platform location. Earlier workers found no changes in spatial reference memory and working memory in Morris water tank with methamidophos in adult rats [35]. The acquisition was not affected with CPS in mice [36] and in Pb treatment [37]. Parathion significantly increased learning, opining that adverse effects were seen only at higher doses. Repeated exposure to OPs could decrease behavioral effects due to tolerance resulting from compensatory changes in central nervous system. ACh in small doses is neurotrophic promoting neural cell differentiation, but as the dose of OP is increased, increase in ACh would cause down-regulation of muscarinic receptors [38]. The differences in neurobehavioral measures depended on the pesticide, specific end point and the time of measurement. Literature contrary to the results obtained shows decreased memory both while treatment [39] after treatment [40] and could affect either repeated acquisition.

Lead-induced (PND1-21) deficits in spatial learning were reversed when rats were raised in the enriched environment [41]. Gilbert *et al*. [42] exposed rats to 0.2% PbAc, as in the present study, from GD16to PND21 and reported reduced neurogenesis but no impairment of spatial learning in MWM. Long-term Pb exposure was not associated with permanent brain dysfunction at a blood Pb concentration of 2 mM and some of the neuropsychological deficits seen could be due to attention deficit disorder. The results in the present study agree with the above-mentioned investigators that although learning and memory might have been affected, that could have been reversed or alleviated in the adult stage.

Thyroid gland of dams after PND21, i.e., the day of weaning was subjected to histopathology. The interlobular stroma was poorly defined. The follicular cells were flattened. Among the treatment groups, MMI, MCP and PbAc microfollicles were visible, had an irregular shape, absolutely empty with no colloid material and dense stroma appeared between the lobules (Figures-1 and 2). MMI is an anti-thyroid compound and would cause primary hypothyroidism as evidenced by its carbethoxy derivative carbimazole in first generation pups [43].

Rat cortex (neocortex, parietal, and frontal) develops between GD 14 and 20. The cerebral cortical layers showed a decrease in cellularity in all the treated groups as compared to control (Figures-3 and 4). This proves that the treatment affected cortical neurogenesis. Further, when a xenobiotic interferes with neurogenesis, the precursors are killed without replacement. Such a gap would be filled by abnormal cell migration and differentiation.

The same was the case with the molecular, Purkinje, and granular layers of the cerebellum. In MCP-treated group, the Purkinje cell layer was in total disarray. Focal loss of Purkinje cells was seen in the PbAc-treated group (Figures-5 and 6).

Hence, based on the study, it is concluded that MCP might affect neurodevelopment through its thyrotoxic action as evinced by the distorted histology of thyroid, cortex and cerebellum. PbAc too could affect the thyroid, and thus caused developmental anomalies in the brain as evinced by increased T_4_.

Region-specific and neurotransmitter-based evaluation need to be taken up to further understand the mechanisms of neurodevelopment and neurobehavioral toxicity.

## Conclusion

MCP and lead might have affected the development of cerebrum and cerebellum via thyroid disruption leading to developmental neurotoxicity.

## Authors’ Contributions

The present article was part of BKK's Ph.D. research work and designed the experimental protocol. AGR, AVK and SSYHQ made critical suggestions in conducting the experiment and critically reviewed the manuscript. PSK drafted and revised the manuscript. All authors read and approved the final manuscript.
